# Supplementary material for: Topotactic Phase Transition in Epitaxial La0.7Sr0.3MnO3‐δ Films Induced by Oxygen Getter Assisted Thermal Annealing
Source: Small. 2025 Dec 9;22(7):e10577. doi: 10.1002/smll.202510577 (PMC12862449; doi:10.1002/smll.202510577)
Supplement: Supplementary file 1 — Supporting Information [file SMLL-22-e10577-s001.docx]

Supporting Information:

Topotactic phase transition in epitaxial La_0.7_Sr_0.3_MnO_3-δ_ films induced by oxygen getter assisted thermal annealing

*Chenyang Yin^1,2*^, Lei Cao^3,2^, Xue Bai*^4^*, Suqin He^5,2^, Hengbo Zhang^2^, Tomáš Duchoň^6^,
Felix Gunkel^5^, Yunxia Zhou^7^, Mao Wang^7,8^, Anton Kaus^5^, Janghyun Jo*^4^*,* *Rafal E. Dunin-Borkowski*^4^*, Shengqiang Zhou^7^, Thomas Brückel^2^, Oleg Petracic^2,1*^*

[1] Faculty of Mathematics and Natural Sciences, Heinrich Heine University Düsseldorf, 40225, Düsseldorf, Germany

[2] Jülich Centre for Neutron Science (JCNS-2), JARA-FIT, Forschungszentrum Jülich GmbH, 52428, Jülich, Germany

[3] School of Advanced Materials, Peking University, Shenzhen Graduate School, Shenzhen, 518055, China

[4] Ernst Ruska-Centre for Microscopy and Spectroscopy with Electrons (ER-C), JARA-FIT, Forschungszentrum Jülich GmbH, 52428, Jülich, Germany

[5] Peter Grünberg Institut (PGI-7), JARA-FIT, Forschungszentrum Jülich GmbH, 52428, Jülich, Germany

[6] Peter Grünberg Institut (PGI-6), JARA-FIT, Forschungszentrum Jülich GmbH, 52428, Jülich, Germany

[7] Institute of Ion Beam Physics and Materials Research, Helmholtz-Zentrum Dresden-Rossendorf (HZDR), 01328, Dresden, Germany

[8] Laboratory of Micro-Nano Optics, College of Physics and Electronic Engineering, Sichuan Normal University, Chengdu, 610101, China

*Corresponding authors:

Oleg Petracic

o.petracic@fz-juelich.de

Chenyang Yin

c.yin@fz-juelich.de

**S1:**

Sample ID, post-treatment and sample label information in the manuscript is illustrated in Table S1.1.

Table S1.1: Sample ID, post-treatment and sample label in the manuscript.

| **Sample ID** | **Post-treatment (Al-assisted thermal vacuum annealing)** | **Sample label** |
| --- | --- | --- |
| SP578raw |  | PV-LSMO |
| SP578-1 | 350 °C 12h, with 0.5g Al | E-PV350 |
| SP578-2 | 400 °C 12h, with 0.5g Al | BM400 |
| CYPLD002-4-1 | 400 °C 12h, with 0.5g Al | PLD400 |

Additional sample ID, post-treatment and sample label information in the supporting information is illustrated in Table S1.2.

Table S1.2: Additional sample ID, post-treatment and sample label.

| **Sample ID** | **Post-treatment (Al-assisted thermal vacuum annealing)** | **Sample label** |
| --- | --- | --- |
| SP570-1 | 300 °C 12h, with no Al |  |
| SP570-2 | 300 °C 12h, with 0.5g Al |  |
| SP570-3 | 300 °C 12h, with 1g Al |  |
| SP570-5 |  | PV-LSMO-RE |
| SP578-3 | 450 °C 12h, with 0.5g Al |  |
| R3 |  |  |
| PB01 | 550 °C 20h, with 0.5g Al |  |
| CYPLD002raw |  | PLD-PV |
| SP601-1 |  |  |
| SP601-2 | 400 °C 6h, with 1g Al, ca. 200 cm^3^ tube |  |
| SP601-3 | 400 °C 12h, with 1g Al, ca. 200 cm^3^ tube |  |
| SP601-4 | 450 °C 12h, with 1g Al, ca. 200 cm^3^ tube |  |

**S2:**

The XRR patterns were fitted using the software GenX. The three-layer-model consists of one top LSMO layer (0.2-3.0nm), one middle LSMO layer (main layer) and one bottom LSMO layer (0.2-3.0nm) on bulk STO substrate (Figure S2a). The top layer and bottom layer describe the air-film interface and the film-substrate interface, respectively. The four-layer-model comprises one top LSMO layer, two main LSMO layers in the middle and one bottom LSMO layer (Figure S2b). When the fitted roughness is larger than the thickness implying that the film is not continuous but consists of structures such as island-like particles (e.g. the LSMO top layer). The total thickness only considers the thickness of LSMO main layers. This total thickness is used in the following magnetization normalization. See Table S2 for partial fitting results. In the RBS fitting, the thickness is fitted by the software SIMNRA.


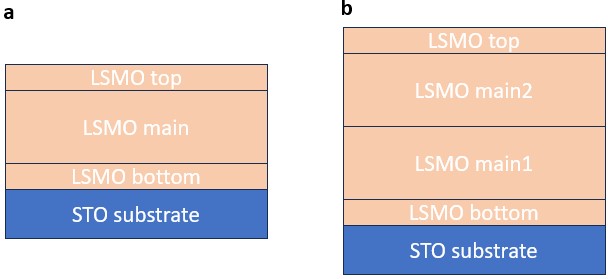


Figure S2. (a) Illustration of the three-layer-model for XRR fitting. (b) Illustration of the four-layer-model for XRR fitting.

Table S2.XRR fitting results of the as-prepared and annealed films

|  | **PV-LSMO** | **E-PV350** | **BM400** |
| --- | --- | --- | --- |
| **LSMO top layer thickness**  **[nm]** | 0.2±0.5 | 2.0±0.5 | 0.2±0.5 |
| **LSMO**  **top layer**  **roughness**  **[nm]** | 0.7±0.5 | 1.2±0.5 | 6.2±0.5 |
| **LSMO**  **top layer**  **SLD**  **[**${{10}^{-5}Å}^{-2}$**]** | 1.07±0.05 | 0.28±0.05 | 1.94±0.05 |
| **LSMO**  **main layer (2) thickness**  **[nm]** |  |  | 12.1 ±0.5 |
| **LSMO**  **main layer (2)**  **roughness**  **[nm]** |  |  | 2.2±0.5 |
| **LSMO**  **main layer (2) SLD**  **[**${{10}^{-5}Å}^{-2}$**]** |  |  | 2.80±0.05 |
| **LSMO**  **main layer (1) thickness**  **[nm]** | 41.9±0.5 | 37.5±0.5 | 31.5±0.5 |
| **LSMO**  **main layer (1)**  **roughness**  **[nm]** | 2.1±0.5 | 2.6±0.5 | 2.5±0.5 |
| **LSMO**  **main layer (1) SLD**  **[**${{10}^{-5}Å}^{-2}$**]** | 4.64±0.05 | 4.28±0.05 | 3.64±0.05 |
| **LSMO**  **bottom layer thickness**  **[nm]** | 1.3±0.5 | 0.6±0.5 | 0.3±0.5 |
| **LSMO**  **bottom layer roughness**  **[nm]** | 0.8±0.5 | 1.4±0.5 | 2.7±0.5 |
| **LSMO**  **bottom layer SLD**  **[**${{10}^{-5}Å}^{-2}$**]** | 4.24±0.05 | 4.67±0.05 | 4.48±0.05 |
| **LSMO total thickness (main layers)**  **[nm]** | 41.9±0.5 | 37.5±0.5 | 43.6±0.7 |

**S3:**

The XRD patterns of the films annealed at 300 °C for 12h with different Aluminum content are shown in Figure S3.1. With increasing Aluminum content, a continuous left-shifted thin film Bragg peak position is observed. This means more oxygen vacancies are introduced in the film with the increased mass of the oxygen getter material during annealing i.e. the polished Aluminum foil. Thus, the improvement of vacuum annealing efficiency by using the polished Aluminum foil is verified.


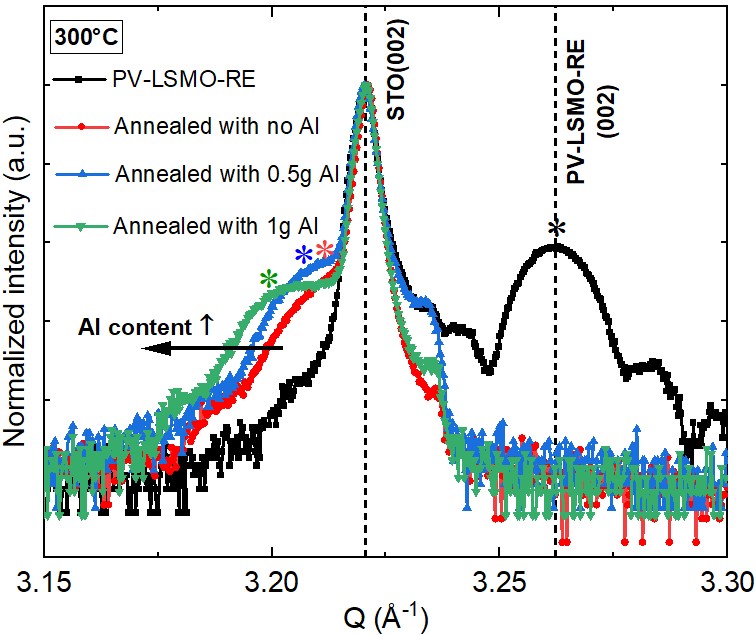


Figure S3.1. XRD patterns of the samples annealed at 300 °C for 12h with different Aluminium content. With no Aluminium (red), with 0.5g Aluminium (blue) and with 1g Aluminium (green).

The XRD pattern of the HOPSD thin-film sample annealed at 450 °C for 12 h is shown in Figure S3.2a. Compared with the sample annealed at 400 °C for 12 h (BM400), the normalized Bragg reflection of the BM (008) peak exhibits a noticeably lower intensity. This indicates a reduction in the thickness of the crystalline BM layer and a corresponding increase in the near-surface amorphous layer, which is further supported by the XRR results (Figures S3.2b and S3.2c, Table S3). In particular, the near-surface low-SLD region becomes thicker and extends deeper into the film (highlighted by the blue arrow in Figure S3.2c).

The higher annealing temperature (450 °C) promotes oxygen diffusion, leading to faster oxygen loss and thus a higher topotactic transformation rate. The observed thickening of the near-surface amorphous layer suggests that part of the BM phase is converted into an amorphous phase upon further oxygen removal, corresponding to the deconstruction of the lattice framework. This interpretation is further supported by the film thickness-dependent topotactic phase transition shown in Figure S8.2. Based on these results, a schematic illustration has been added in Figure S3.3, showing that a fully amorphous layer is expected at higher annealing temperatures (> 450 °C). We believe that this transformation process also occurs during annealing at the same temperature for longer durations. The corresponding evolution pathway is summarized schematically in Figure S3.4.

The proposed evolution pathway is as follows (Figure S3.4):

(1) The surface region undergoes severe oxygen loss due to the short diffusion path to the surface, leading to the rapid formation of a near-surface amorphous layer. This surface modification has already been observed in the E-PV350 sample (Figures 2b and 2e) before the BM phase forms.

(2) The region below the surface remains as a single crystalline phase, as reflected by the SLD profile of E-PV350 (red curve in Figure 1e).

(3) Once the overall film reaches the BM phase, further oxygen removal causes a breakdown of the lattice structure, accelerating the formation of the amorphous layer.

Regarding surface segregation, we believe it occurs concurrently with the amorphous layer formation, since the destruction of the lattice facilitates cation diffusion and segregation.


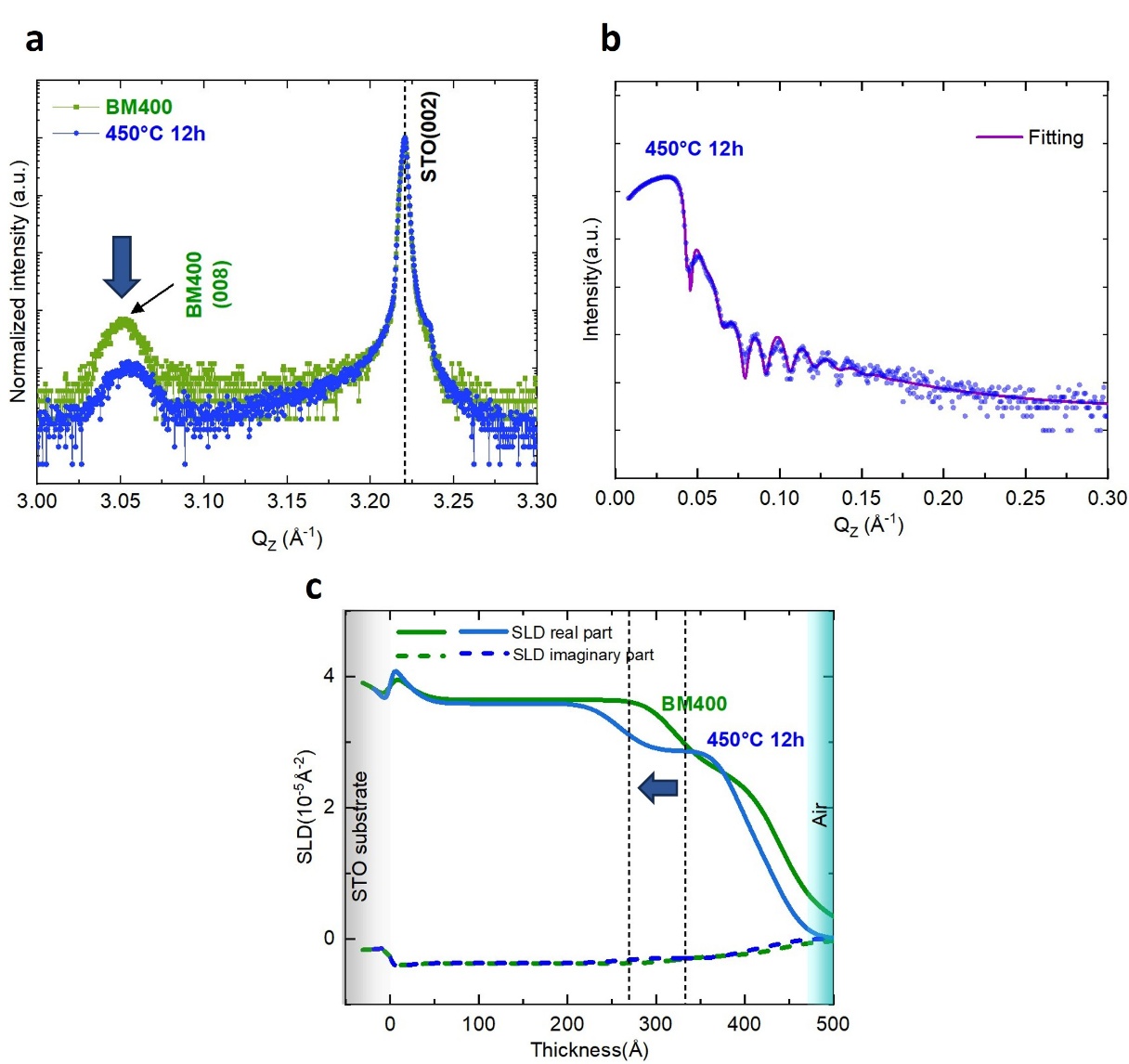


Figure S3.2. (a) XRD patterns of the annealed LSMO films, annealed for 12 h at 400 °C (green) and at 450 °C (blue), respectively. (b) XRR pattern of the sample annealed at 450 °C for 12 h. The fitting curve obtained using the GenX software is shown in purple. Pattern fitted via a four-layer model (Figure S2b). (c) Real (Re.) and imaginary (Im.) SLD profiles as derived from the XRR pattern fitting.

Table S3. XRR fitting results of the annealed film

|  | **450°C 12h annealed** |
| --- | --- |
| **LSMO top layer thickness**  **[nm]** | 4.7±0.5 |
| **LSMO**  **top layer**  **roughness**  **[nm]** | 2.7±0.5 |
| **LSMO**  **top layer**  **SLD**  **[**${{10}^{-5}Å}^{-2}$**]** | 1.75±0.05 |
| **LSMO**  **main layer (2) thickness**  **[nm]** | 12.9 ±0.5 |
| **LSMO**  **main layer (2)**  **roughness**  **[nm]** | 2.0±0.5 |
| **LSMO**  **main layer (2) SLD**  **[**${{10}^{-5}Å}^{-2}$**]** | 2.86±0.05 |
| **LSMO**  **main layer (1) thickness**  **[nm]** | 25.3±0.5 |
| **LSMO**  **main layer (1)**  **roughness**  **[nm]** | 2.7±0.5 |
| **LSMO**  **main layer (1) SLD**  **[**${{10}^{-5}Å}^{-2}$**]** | 3.59±0.05 |
| **LSMO**  **bottom layer thickness**  **[nm]** | 0.5±0.5 |
| **LSMO**  **bottom layer roughness**  **[nm]** | 2.4±0.5 |
| **LSMO**  **bottom layer SLD**  **[**${{10}^{-5}Å}^{-2}$**]** | 4.66±0.05 |
| **LSMO total thickness (main layers)**  **[nm]** | 38.2±0.7 |


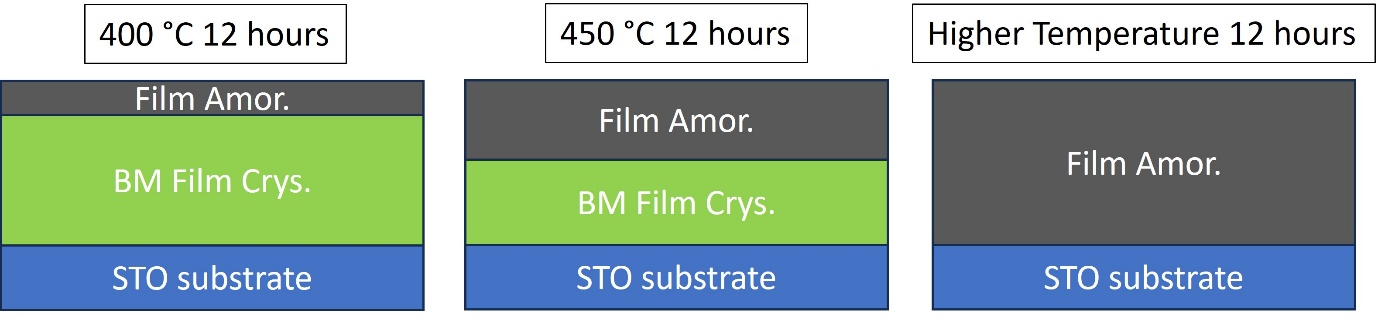


Figure S3.3. Schematic illustration showing the increased amorphous layer resulting from higher annealing temperatures.


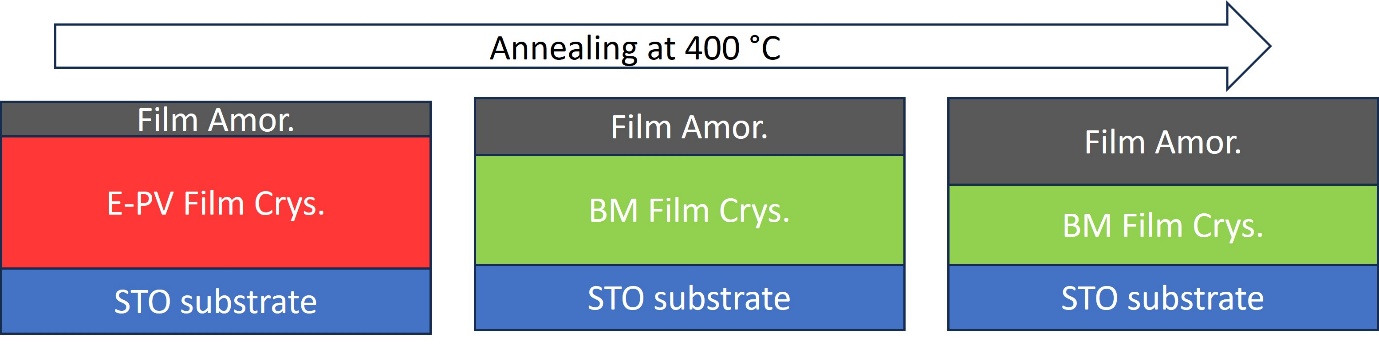


Figure S3.4. Schematic illustration of annealing experiments conducted at 400 °C for different durations.

**S4:**

The sample stability in air is shown in Figure S4. BM400 shows long-term stability after air exposure. The continuous right-shifted thin film Bragg peak position of E-PV350 indicates a gradual oxygen retake after air exposure. The corresponding stages when specific measurements were performed are marked.


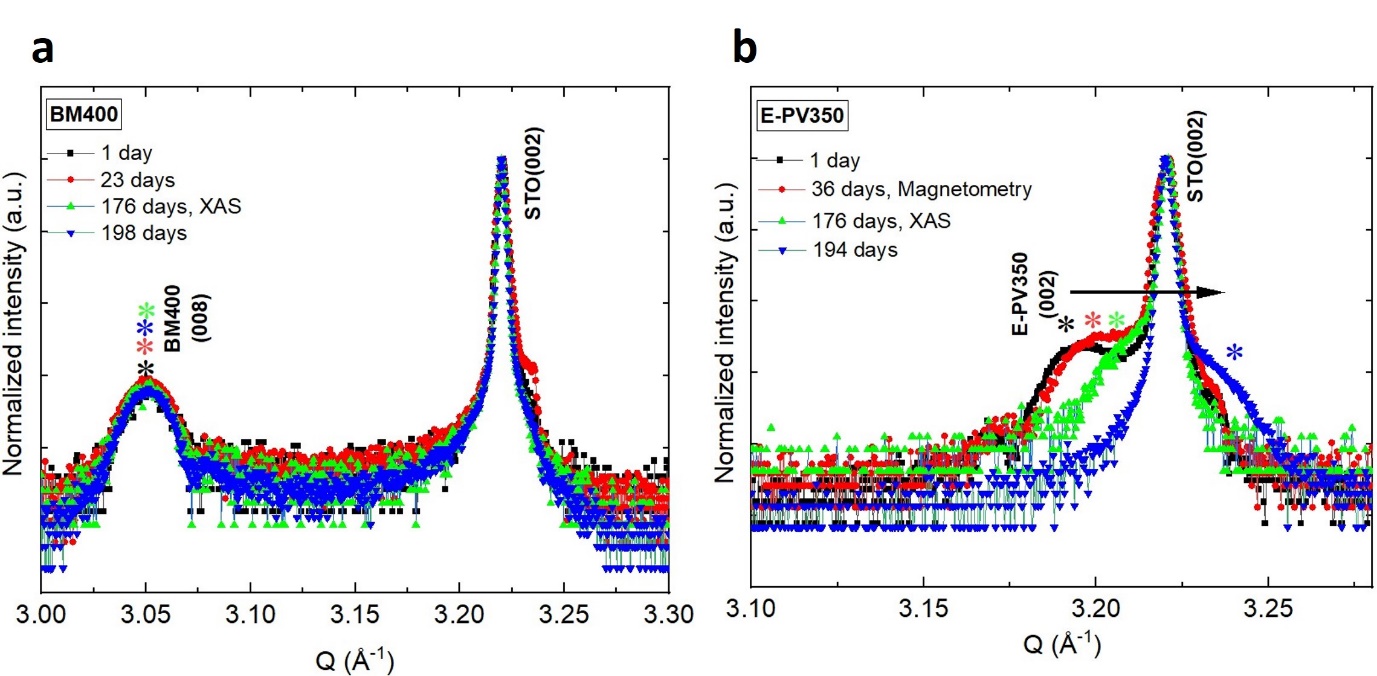


Figure S4. XRD patterns of the annealed films, measured after air exposure for different times. (a)BM400. (b) E-PV350.

**S5:**

Figure S5 demonstrates the field-dependent ZFC FC curves of the 400°C 12h annealed film (BM400). The ZFC FC curves measured at 10mT show a potential intersection at a temperature >400K indicating a probably novel magnetic ordering.


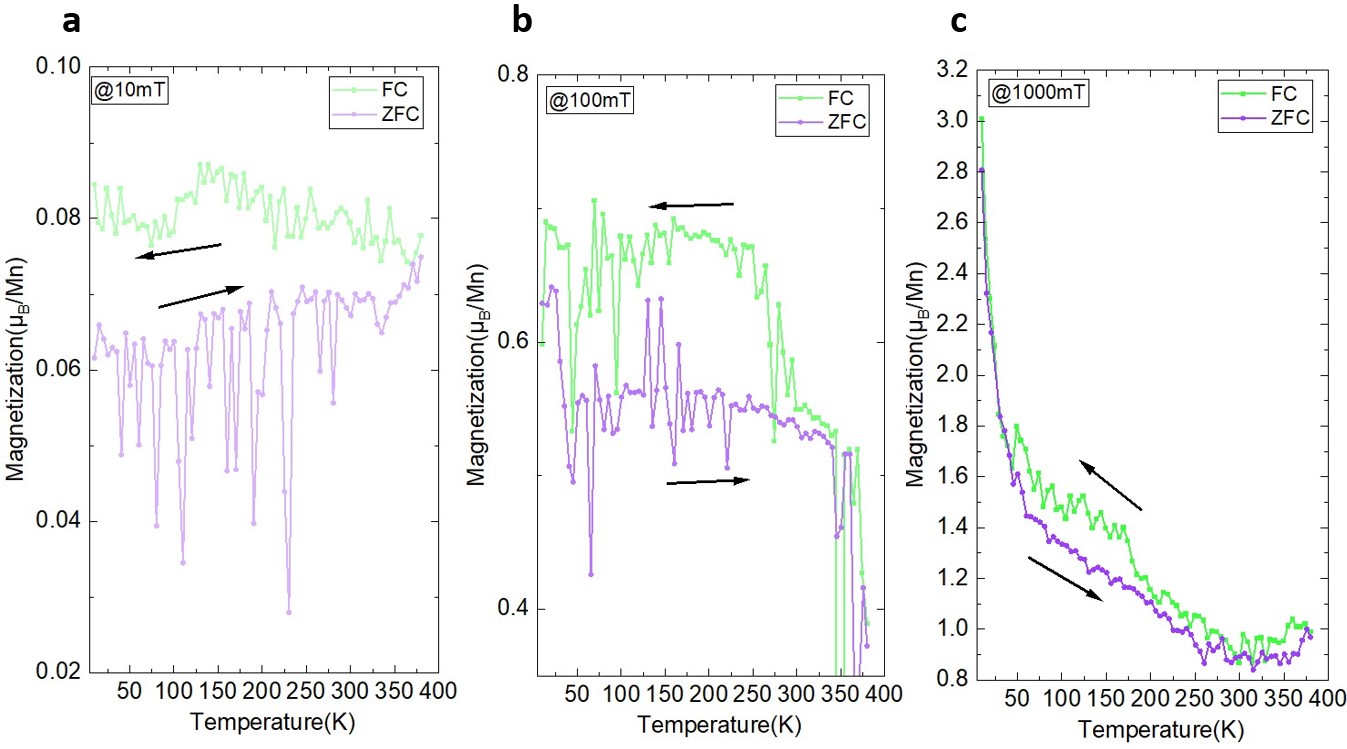


Figure S5. The field-dependent ZFC FC curves of 400°C 12h annealed film (BM400). (a) Measured at 10mT. (b) Measured at 100mT. (c) Measured at 1000mT.

**S6:**

Figure S6 illustrates the raw thin film magnetic data without normalization. The kinks between 200K and 300K in Figure S6b are due to the usual “zero-crossing” problem in magnetometry.


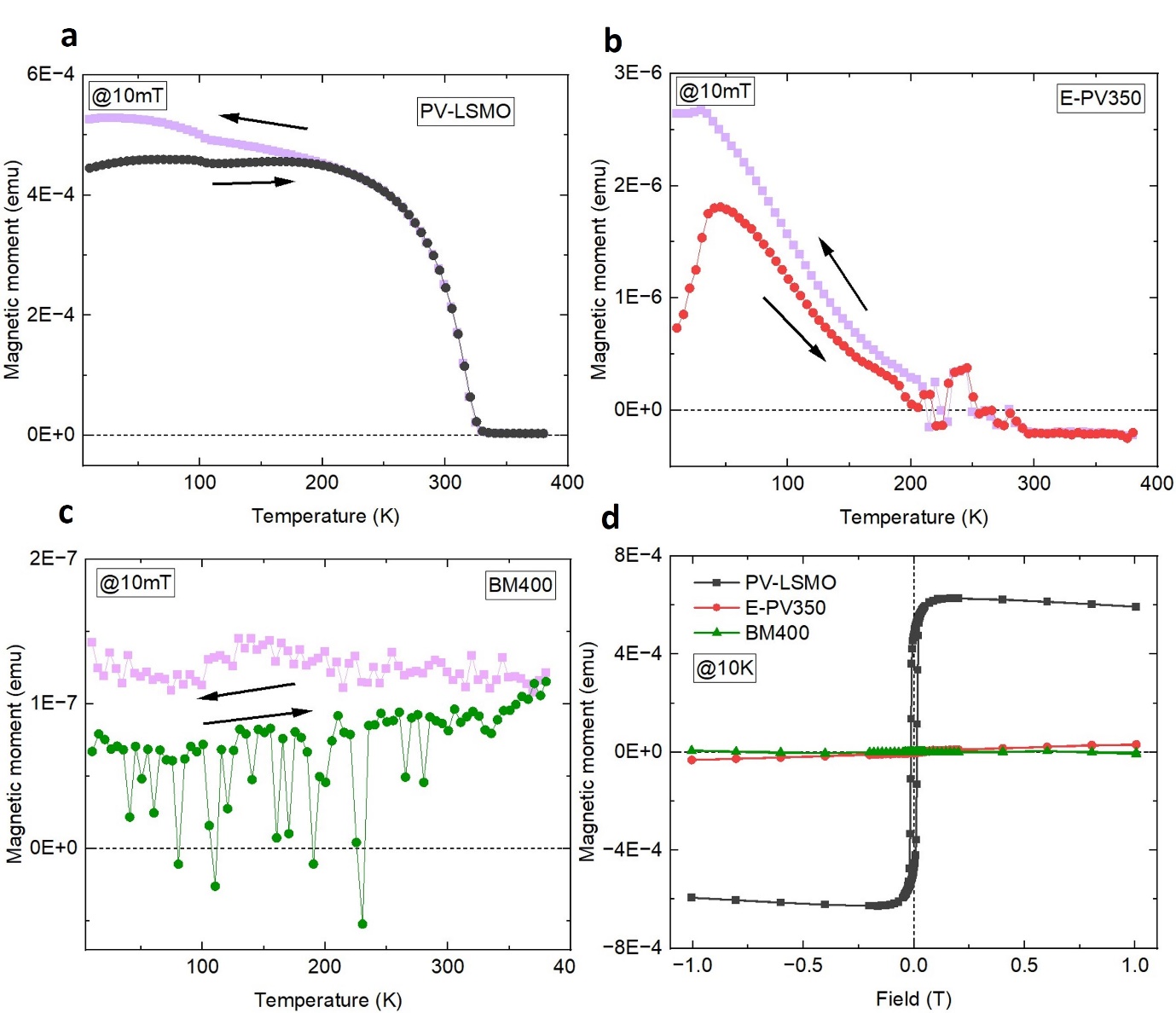


Figure S6. Raw magnetic data. (a) ZFC FC curves of the PV-LSMO, measured at 10mT. (b) ZFC FC curves of the E-PV350, measured at 10mT. (c) ZFC FC curves of the BM400, measured at 10mT. (d) Hysteresis loops of the as-prepared and annealed thin films measured at 10K.

**S7:**

The powder sample is prepared via ball-milling using a stoichiometric La_0.7_Sr_0.3_MnO_3_ sputtering target. SEM confirms that the powder sample exhibits an average size of ca. 3 μm. SEM images were taken using the JSM-6510 series instrument at the institute JCNS-1. The XRD patterns of the powder sample before (R3, as-prepared) and after annealing (PB01, annealed) are shown in Figure S7. Data is collected using a powder diffractometer (Huber Imaging Plate Guinier Camera G670 and X-ray Cu source). After performing Al-assisted thermal vacuum annealing at 550 °C for 20 hours, the BM phase is achieved. This is confirmed by comparing with the ICSD data (ICSD-50717 and ICSD-166141). The tube size and vacuum condition used for annealing are identical to the thin film samples. 50 mg of powder is used for annealing.


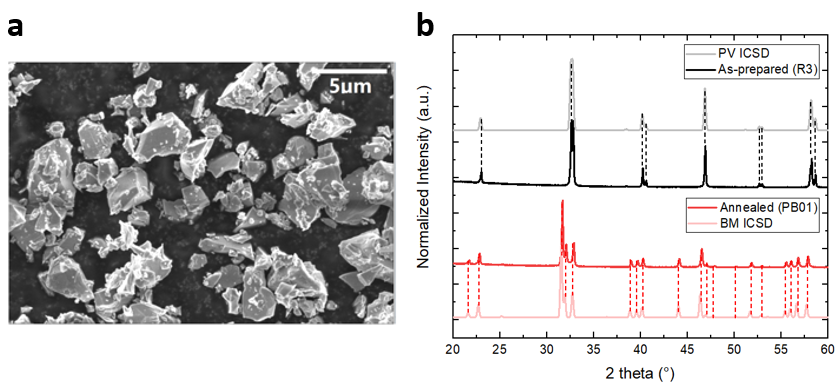


Figure S7. (a) Scanning Electron Microscope (SEM) image of the as-prepared powder sample. (b) XRD patterns of the powder sample before and after annealing.

**S8:**

The surface morphology of the as-prepared film grown using PLD is characterized via AFM and shown in Figure S8a, which exhibits a very good surface quality and an RMS roughness of ca. 0.25 nm. The crystal structure of the film before and after Al-assisted thermal vacuum annealing is probed via XRD using a D8 ADVANCE and illustrated in Figure S8b. The superlattice peaks confirmed a successful topotactic phase transition from PV to BM.

Before the STEM measurements, Pt is deposited to enhance surface conductivity thus avoiding charging. The sulfur and chlorine are from the Pt deposition via chemical vapor deposition method (organic). Carbon is introduced due to carbon contamination during the STEM measurements.


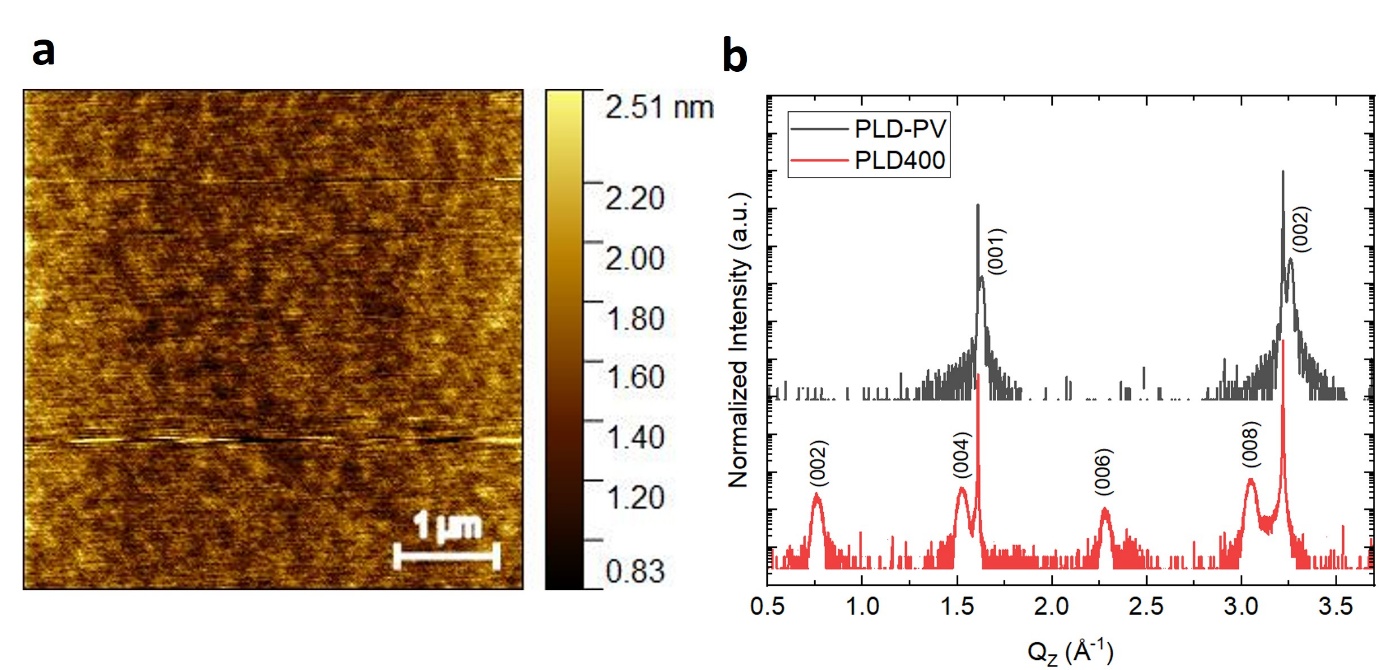


Figure S8.1 (a) AFM image of the as-prepared film grown via PLD. (b) XRD patterns of the film before and after annealing.

The XRR patterns of the PLD sample before and after annealing are shown in Figure S8.2a. A three-layer model (Figure S2a) was used to fit the as-prepared PLD sample (PLD-PV), while a four-layer model (Figure S2b) was applied to the annealed PLD sample (PLD400). The same fitting strategy was employed for the HOPSD thin film samples, i.e., the three-layer model for PV-LSMO and the four-layer model for BM400. All fittings were performed using the GenX software. The as-prepared PLD sample (PLD-PV) exhibits a thickness of 26 nm (Table S8), which is thinner than that of the HOPSD sample (LSMO-PV, 42 nm, Table S2). A comparison between the HOPSD (42 nm) and PLD (26 nm) samples before and after annealing is shown in Figure S8.2b. The thinner PLD sample displays a distinct SLD profile compared with the HOPSD sample, characterized by a lower plateau region in the SLD curve (indicated by the purple arrow in Figure S8.2b).

According to the STEM results (Figure 4a) of the PLD400 sample, the corresponding phase distribution along the depth direction has been marked in the SLD profile (Figure S8.2c). The BM-phase region exhibits a gradual SLD transition, which can be attributed to the limited thickness (ca. 14 nm) of the BM region (the “bottom” and “LSMO main1” layers) and the curved interface between the BM and amorphous layers (Figure S8.3). The extended near-surface amorphous layer contributes to the formation of the plateau in the SLD profile. When the same XRR fitting model is applied to the thicker HOPSD film, the BM region (“bottom” and “LSMO main1”) becomes thicker, resulting in a more pronounced plateau in the SLD profile (Figure S8.2d), while the near-surface amorphous layer is reduced. Based on these results, we infer that thinner films (e.g., the PLD sample) undergo a faster topotactic phase transition under the same annealing conditions, due to the shorter bottom-to-surface oxygen diffusion path. This further suggests that, once the BM phase is formed, continued annealing (i.e., additional oxygen removal) will eventually transform the BM phase into an amorphous structure.


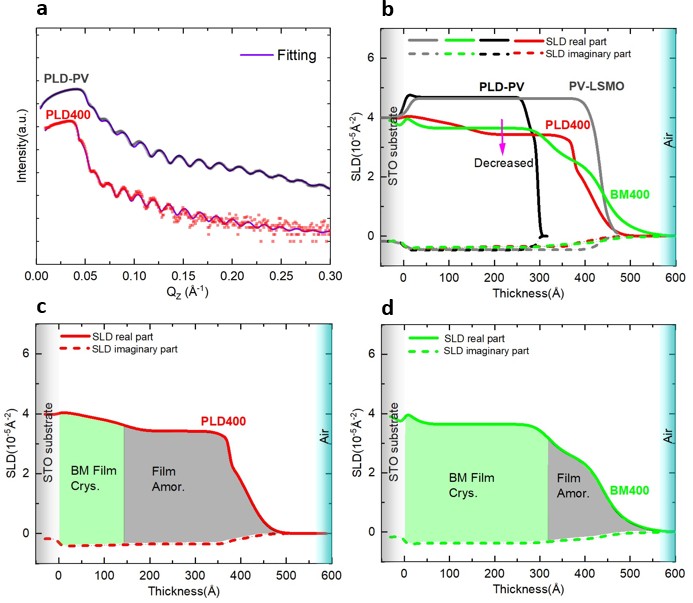


Figure S8.2. (a) XRR patterns of the as-prepared and annealed PLD samples. The fitting curves obtained using the GenX software are shown in purple. (b) Real (Re.) and imaginary (Im.) SLD profiles of the as-prepared and annealed PLD samples derived from the fitting. For comparison, the as-prepared and annealed HOPSD samples are also included. (c, d) SLD profiles of the annealed PLD and HOPSD samples extracted from (b). The crystalline BM phase region (green zone) and the near-surface amorphous region (grey zone) are highlighted.

Table S8.XRR fitting results of the as-prepared and annealed PLD films

|  | **PLD-PV** | **PLD400** |
| --- | --- | --- |
| **LSMO top layer thickness**  **[nm]** | 2.3±0.5 | 4.0±0.5 |
| **LSMO**  **top layer**  **roughness**  **[nm]** | 0.4±0.5 | 3.5±0.5 |
| **LSMO**  **top layer**  **SLD**  **[**${{10}^{-5}Å}^{-2}$**]** | 3.27±0.05 | 2.66±0.05 |
| **LSMO**  **main layer (2) thickness**  **[nm]** |  | 23.1±0.5 |
| **LSMO**  **main layer (2)**  **roughness**  **[nm]** |  | 0.4±0.5 |
| **LSMO**  **main layer (2) SLD**  **[**${{10}^{-5}Å}^{-2}$**]** |  | 3.42±0.05 |
| **LSMO**  **main layer (1) thickness**  **[nm]** | 26.0±0.5 | 8.9±0.5 |
| **LSMO**  **main layer (1)**  **roughness**  **[nm]** | 0.9±0.5 | 3.3±0.5 |
| **LSMO**  **main layer (1) SLD**  **[**${{10}^{-5}Å}^{-2}$**]** | 4.69±0.05 | 3.80±0.05 |
| **LSMO**  **bottom layer thickness**  **[nm]** | 1.3±0.5 | 5.5±0.5 |
| **LSMO**  **bottom layer roughness**  **[nm]** | 1.1±0.5 | 3.3±0.5 |
| **LSMO**  **bottom layer SLD**  **[**${{10}^{-5}Å}^{-2}$**]** | 4.89±0.05 | 4.06±0.05 |
| **LSMO total thickness (main layers)**  **[nm]** | 26.0±0.5 | 32.0±0.5 |


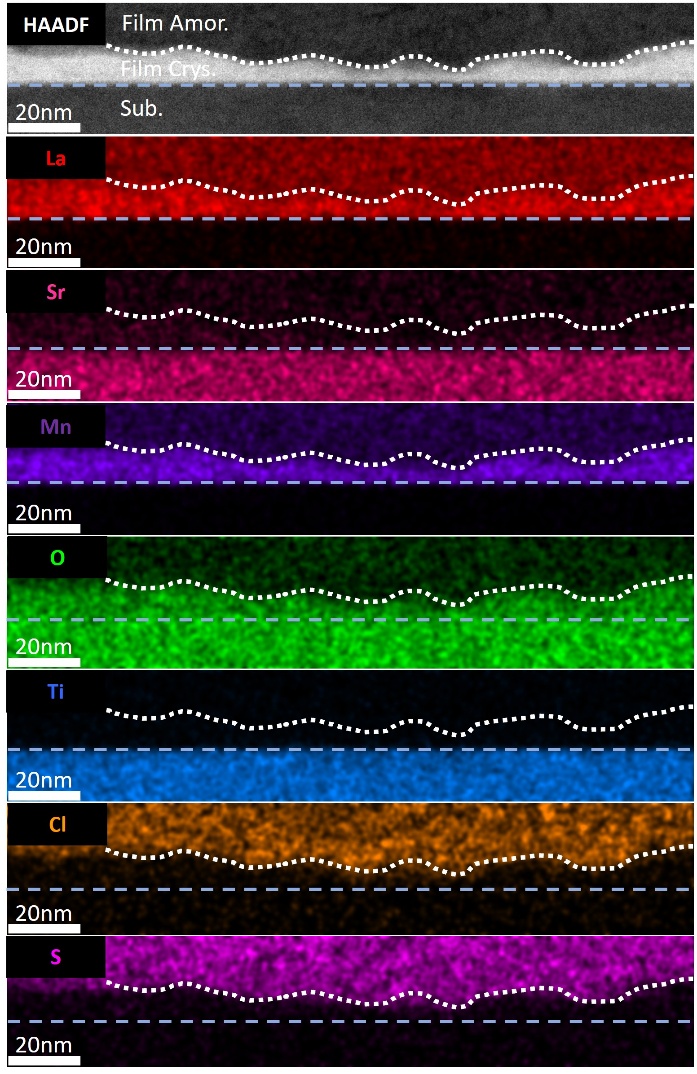


Figure S8.3. EDS elemental mapping of a larger region of the annealed PLD sample (PLD400). The white dotted line marks the interface between the crystalline BM phase and the amorphous layer.

To further clarify the structural features at different depths, additional discussions are provided based on Figure S8.4. In the zoom-in HAADF image of the Mn-segregated region (Figure S8.4b), weak stripe-like contrasts are observed, indicating the presence of crystalline order within this region. According to the EDS elemental mapping (Figure 4a), Mn and S are the dominant elements in this segregated area. Given that HAADF imaging is less sensitive to light elements (e.g., S and O), these weak stripes are attributed primarily to Mn atoms, suggesting the formation of an MnS phase. The FFT of the segregated region (Figure S8.4d) indicates that the phase corresponds to α-MnS.

We infer that the MnS phase was formed through the reduction of manganese oxides (MnO_X_) by sulfur (S) during the organic CVD Pt deposition process (Section S8), which occurred prior to the STEM measurements. The manganese oxides (MnO_X_) likely originated from the oxidation of exsolved metallic Mn upon air exposure [1]. Similar metal exsolution phenomena from perovskite oxides have been reported in related systems [2-4].

The proposed formation process can be summarized as follows:

(1) Mn exsolution occurs concurrently with the formation of the amorphous layer.

(2) Due to the high chemical reactivity of Mn, the exsolved metal oxidizes in air to form MnO_X_.

(3) During the organic CVD process, sulfur reduces MnO_X_, leading to the formation of crystalline MnS.


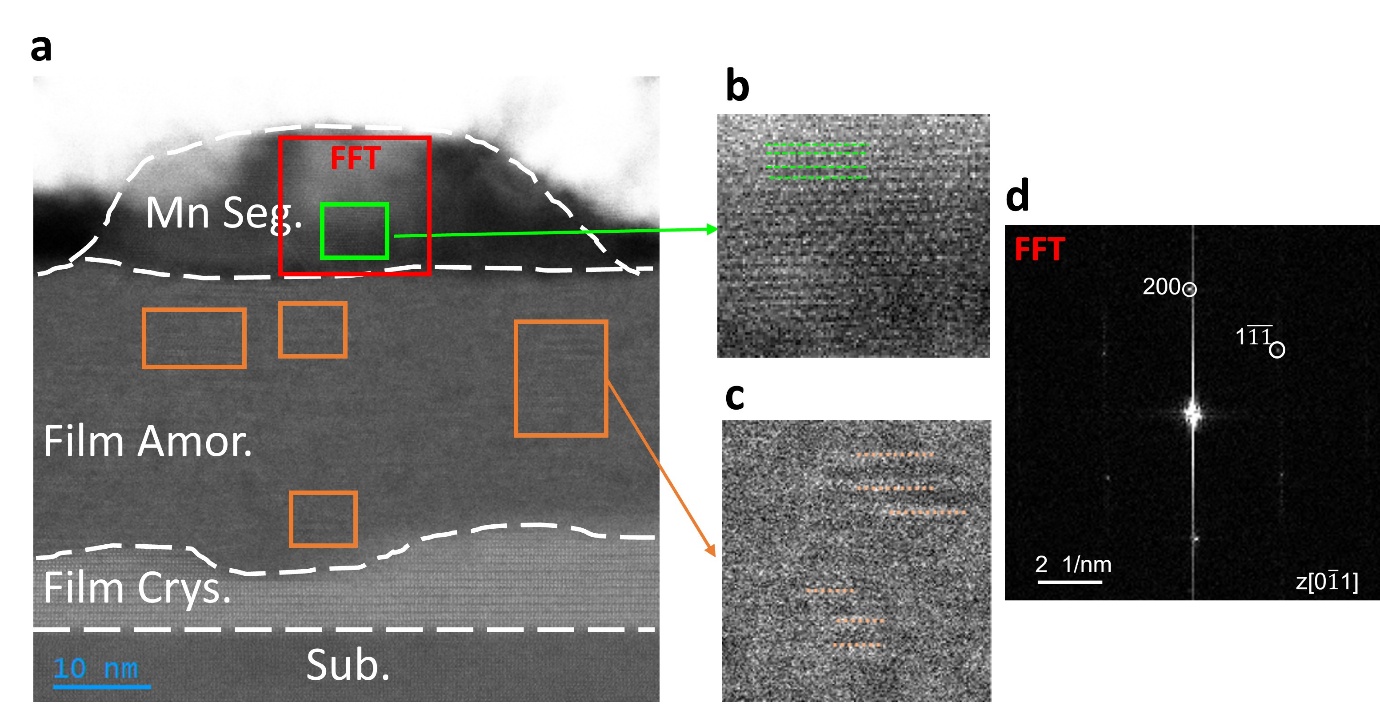


Figure S8.4. (a) HAADF image of the annealed PLD sample (PLD400). (b) Zoom-in of the manganese segregated region. The green lines denote the atomic planes. (c) Zoom-in of the amorphous region, where several stripe-like features indicate local crystal ordering, as marked by brown lines. (d) Fast Fourier transform (FFT) of the region highlighted by the red box in (a), with diffraction spots indexed to α-MnS indicated.

To address the electric contribution, the low-loss EELS spectra of the PLD sample (PLD400) are presented in Figure S8.5. The low-loss range (0-50 eV) corresponds to the zero-loss and plasmon excitation regions. The EELS signals were integrated over three representative areas (Figure S8.5a): Region 1 (Mn-segregated region), Region 2 (amorphous region), and Region 3 (crystalline BM region). For all three regions, only clear zero-loss peaks were detected, whereas the 5-50 eV energy loss window exhibited very weak and noisy features, preventing a reliable determination of the band gap. According to the literature, α-MnS exhibits semiconducting behavior [5]. The MnO_X_ species (prior to sulfur reduction) can display a wide range of electrical transport characteristics, from insulating MnO to semiconducting MnO_2_ [6]. Elemental Mn (before oxidation, e.g., α-Mn) displays metallic behavior [7]. The amorphous region is expected to exhibit pronounced electron localization due to the loss of long-range lattice order and the increased concentration of oxygen vacancies (Figure 4b), both of which strongly suppress the double-exchange interaction. In this region, only short-range structural features are observed (Figure S8.4a and S8.4c). For the crystalline BM phase region, a very high resistivity is also expected as well, primarily due to strong electron localization arising from super-exchange interactions [8].

To evaluate the magnetic contribution, magnetometry measurements were performed for the same PLD sample (PLD400), as shown in Figure S8.6. These measurements were conducted prior to CVD Pt deposition, thereby excluding any influence from S or Cl. The macroscopic magnetization (Figure S8.6a) reveals a low overall magnetic moment and nearly linear ZFC/FC curves without any pronounced magnetic transitions, similar to the BM phase in the HOPSD sample (BM400). The zoomed-in ZFC/FC curves (Figure S8.6b) display a comparable trend to that of BM400 (Figure S5a) and suggest a possible intersection above 400 K, although the precise magnetic behavior cannot be conclusively determined from the current data.

Based on these observations, the following interpretations are proposed:

(1) The Mn-segregated region (MnO_X_) is expected to exhibit complex magnetic behavior, as it may contain multiple Mn oxide phases such as MnO (antiferromagnetic) [6], Mn_3_O_4_ (ferrimagnetic) [9], and MnO_2_ (antiferromagnetic) [6]. Given the very limited volume of this segregated region, its overall contribution to the bulk magnetic signal is expected to be minimal.

(2) The amorphous region, which contains only short-range structural order (Figure S8.4c), is likely to exhibit diamagnetic or non-magnetic behavior. The absence of long-range lattice order strongly suppresses magnetic interactions.

(3) The crystalline BM phase may exhibit a complex antiferromagnetic ordering, which is the subject of our ongoing neutron powder diffraction studies aimed at resolving the spin structure evolution from the perovskite to the brownmillerite phase.


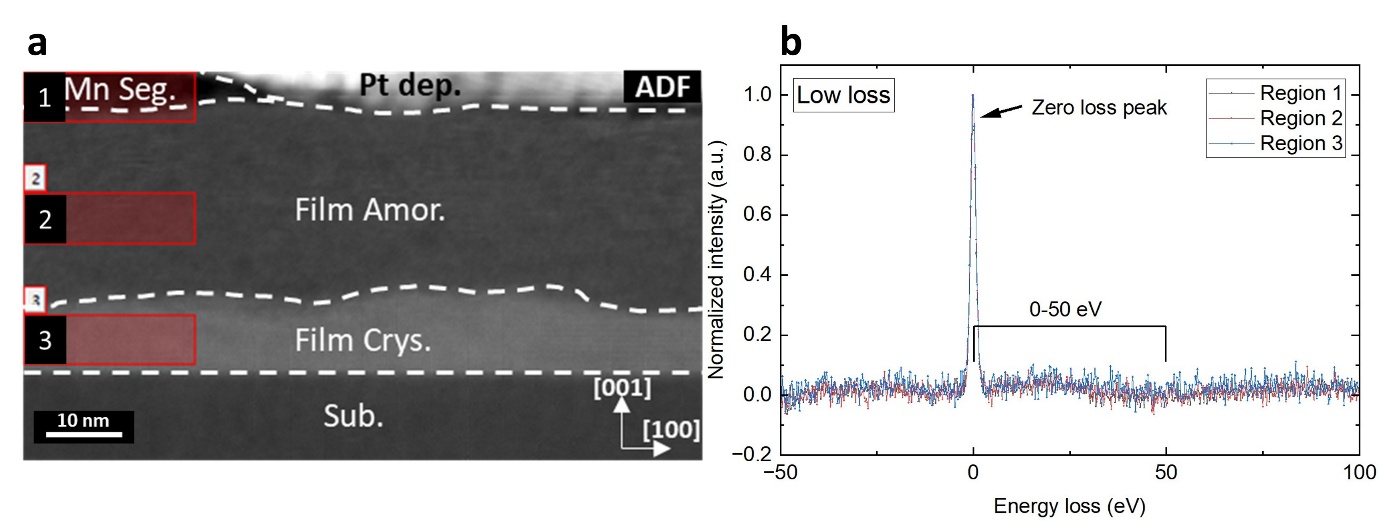


Figure S8.5. (a) ADF image of the annealed PLD sample (PLD400) in the EELS mapping. Three regions are selected. Region 1: Manganese segregated region. Region 2: Amorphous region. Region 3: Crystallized BM-LSMO region. (b) Integrated EELS low-loss signal of the different regions in (a).


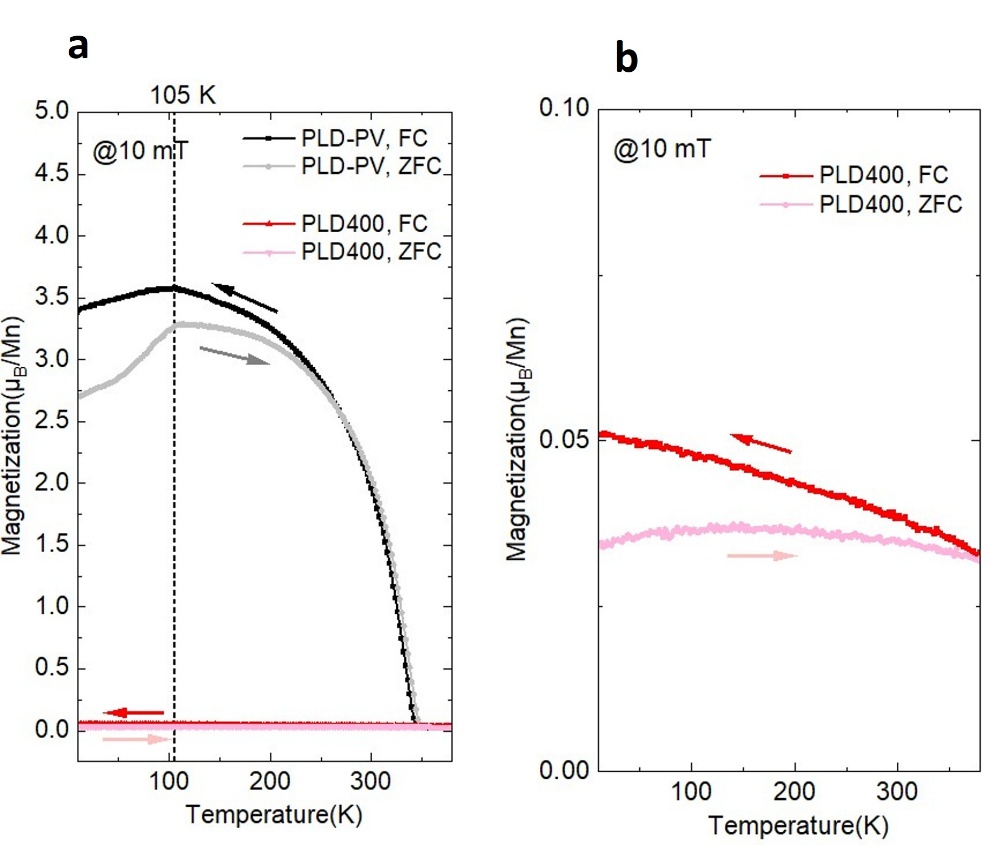


Figure S8.6. (a) Magnetization vs. temperature measurements of the as-prepared and the annealed PLD samples. ZFC and FC curves, measured at 10 mT. (b) Zoom-in of the ZFC and FC curves of the annealed PLD sample.

**S9:**

Here, we include the data for a thicker HOPSD thin film sample (ca. 200 nm). In the XRR pattern, no Kiessig fringes are observed due to the large film thickness, which results in a small *Q*-spacing (Δ*Q* = 2π/*d* ≈ 0.003 Å^-1^) that cannot be resolved (Figure S9.1b). The film thickness was estimated from the deposition time, i.e., 1 h (42 nm, LSMO-PV) to 5 h (ca. 200 nm) using the HOPSD growth method.

The Al-assisted vacuum annealing was then performed on this 200 nm film, and the results are presented in Figure S9.1a. We confirmed that the thicker film requires a higher annealing temperature (450 °C) to reach the BM phase, which can be attributed to the increased bottom-to-surface oxygen diffusion path length. This observation is consistent with the results discussed in Section S8, where the topotactic transformation was compared between the 26 nm PLD and 42 nm HOPSD samples.

Notably, in this 200 nm film, the BM (008) reflection exhibits comparable intensity to the as-prepared PV (002) reflection (grey and red patterns in Figure S9.1a), indicating that no significant amorphous layer is formed. The magnetometry results reveal similar magnetic behavior to those of the thinner HOPSD (BM400) and PLD (PLD400) samples, while the RBS analysis shows that the Mn deficiency observed in the annealed thinner HOPSD film (Figure 3d) disappears in the thicker film, suggesting that cation redistribution and surface segregation are effectively suppressed (Figure S9.1e). These results suggest that optimized annealing parameters can effectively minimize the formation of the amorphous layer and reduce surface segregation.

This 200 nm film was grown by HOPSD using a new sputtering target, which may account for the deviation in Mn stoichiometry observed in the as-prepared sample.


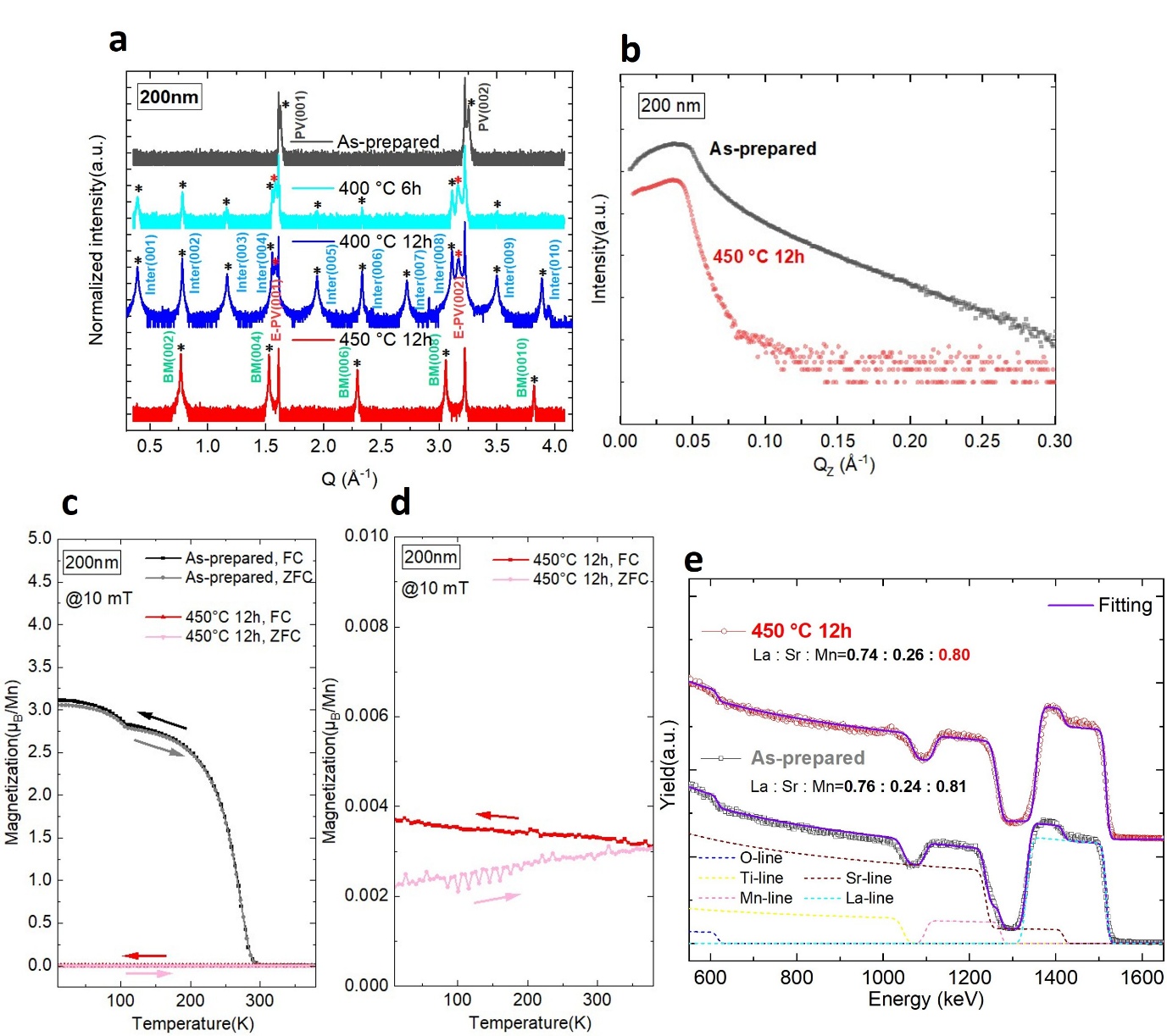


Figure S9.1. (a) XRD patterns of the as-prepared and annealed 200 nm HOPSD samples. (b) XRR patterns of the as-prepared and annealed 200 nm HOPSD samples. (c) Magnetization vs. temperature measurements of the as-prepared and the annealed 200 nm HOPSD samples. ZFC and FC curves, measured at 10 mT. (d) Zoom-in of the ZFC and FC curves of the annealed 200 nm HOPSD samples. (e) RBS spectra of the as-prepared and the annealed 200 nm HOPSD samples.

**Reference:**

1. Foord, J. S., Jackman, R. B., & Allen, G. C., An X-ray photoelectron spectroscopic investigation of the oxidation of manganese. Philosophical Magazine A: Physics of Condensed Matter, Structure, Defects and Mechanical Properties, 1984.49(5): p.657-663.
2. Kwon, O., Sengodan, S., Kim, K., Kim, G., Jeong, H. Y., Shin, J., Ju, Y. W., Han, J. W., & Kim, G., Exsolution trends and co-segregation aspects of self-grown catalyst nanoparticles in perovskites. Nature Communications, 2017.8: p.15967.
3. Weber, M. L., Šmíd, B., Breuer, U., Rose, M. A., Menzler, N. H., Dittmann, R., Waser, R., Guillon, O., Gunkel, F., & Lenser, C., Space charge governs the kinetics of metal exsolution. Nature Materials, 2024.23(3): p.406-413.
4. Jung, S. M., Kim, Y., Yang, D. H., Kim, Y., Kim, K. S., Choe, G., Lee, J., Ihm, K., Choi, S. Y., Son, J., & Kim, Y. T., Low-Temperature Exsolution of Cobalt From Perovskite Nanoparticles via Bead Milling for Enhanced Electrocatalytic Oxygen Evolution Reaction. Advanced Functional Materials, 2025.35(29): p.2506227.
5. Li, N., Zhang, Y., Cheng, R., Wang, J., Li, J., Wang, Z., Sendeku, M. G., Huang, W., Yao, Y., Wen, Y., & He, J., Synthesis and Optoelectronic Applications of a Stable p-Type 2D Material: α-MnS. ACS Nano, 2019.13(11): p.12662-12670.
6. Bhide, V.G., Dani, R.H., Electrical conductivity in oxides of manganese and related compounds. Physica, 1961. 27(9): p.821-826.
7. Desai, P. D., James, H. M., & Ho, C. Y., Electrical Resistivity of Aluminum and Manganese. Journal of Physical and Chemical Reference Data, 1984.13(4): p.1131-1172.
8. Cao, L., Petracic, O., Wei, X. K., Zhang, H., Duchoň, T., Gunkel, F., Koutsioubas, A., Zhernenkov, K., Rushchanskii, K. Z., Hartmann, H., Wilhelm, M., Li, Z., Xie, Y., He, S., Weber, M. L., Veltruská, K., Stellhorn, A., Mayer, J., Zhou, S., Brückel, T., Migration Kinetics of Surface Ions in Oxygen-Deficient Perovskite During Topotactic Transitions. Small, 2021.17(51): p.2104356.
9. Dwight, K., Menyuk, N., Magnetic Properties of Mn_3_O_4_ and the Canted Spin Problem. Physical Review, 1960.119(5): p.1470-1479.
